# Do invasive plants structure microbial communities to accelerate decomposition in intermountain grasslands?

**DOI:** 10.1002/ece3.3608

**Published:** 2017-11-21

**Authors:** Michael R. McTee, Ylva Lekberg, Dan Mummey, Alexii Rummel, Philip W. Ramsey

**Affiliations:** ^1^ MPG Ranch Florence MT USA; ^2^ College of Forestry and Conservation University of Montana Missoula MT USA

**Keywords:** Bacteria, *Bromus tectorum*, *Centaurea stoebe*, ecosystem, *Euphorbia esula*, fungi, home‐field advantage, invasion ecology

## Abstract

Invasive plants are often associated with greater productivity and soil nutrient availabilities, but whether invasive plants with dissimilar traits change decomposer communities and decomposition rates in consistent ways is little known. We compared decomposition rates and the fungal and bacterial communities associated with the litter of three problematic invaders in intermountain grasslands; cheatgrass (*Bromus tectorum*), spotted knapweed (*Centaurea stoebe*) and leafy spurge (*Euphorbia esula*), as well as the native bluebunch wheatgrass (*Pseudoroegneria spicata*). Shoot and root litter from each plant was placed in cheatgrass, spotted knapweed, and leafy spurge invasions as well as remnant native communities in a fully reciprocal design for 6 months to see whether decomposer communities were species‐specific, and whether litter decomposed fastest when placed in a community composed of its own species (referred to hereafter as home‐field advantage–HFA). Overall, litter from the two invasive forbs, spotted knapweed and leafy spurge, decomposed faster than the native and invasive grasses, regardless of the plant community of incubation. Thus, we found no evidence of HFA. T‐RFLP profiles indicated that both fungal and bacterial communities differed between roots and shoots and among plant species, and that fungal communities also differed among plant community types. *Synthesis*. These results show that litter from three common invaders to intermountain grasslands decomposes at different rates and cultures microbial communities that are species‐specific, widespread, and persistent through the dramatic shifts in plant communities associated with invasions.

## 1 INTRODUCTION

Invasion by exotic plants is often associated with higher net primary productivity (NPP) and greater nutrient availability in the soil (Ehrenfeld, 2003; Liao 2008). Many of the mechanisms responsible for these changes occur belowground and can include lack of natural pathogens (Reinhart & Callaway, 2006), increased abundance and activity of symbiotic microbes (Hawkes, Wren, Herman, & Firestone, 2005; Lekberg, Gibbons, Rosendahl, & Ramsey, 2013), and higher mineralization rates of nitrogen (Ehrenfeld, Kourtev, & Huang, 2001; McLeod et al., 2016). Greater nutrient availability in soils largely depends on organic inputs that decomposer communities deliver from litter (Wardle et al., 2004). While decomposer communities are often considered to be functionally redundant (Wardle et al., 2004), potential differences in litter quality among native and invasive plants (Liao et al., 2008) may result in altered decomposition rates and possible shifts in decomposer communities (van der Putten, Klironomos, & Wardle, 2007). The extent to which decomposition rates and the composition of decomposer communities depends on specific invaders is unclear.

**Figure 1:**
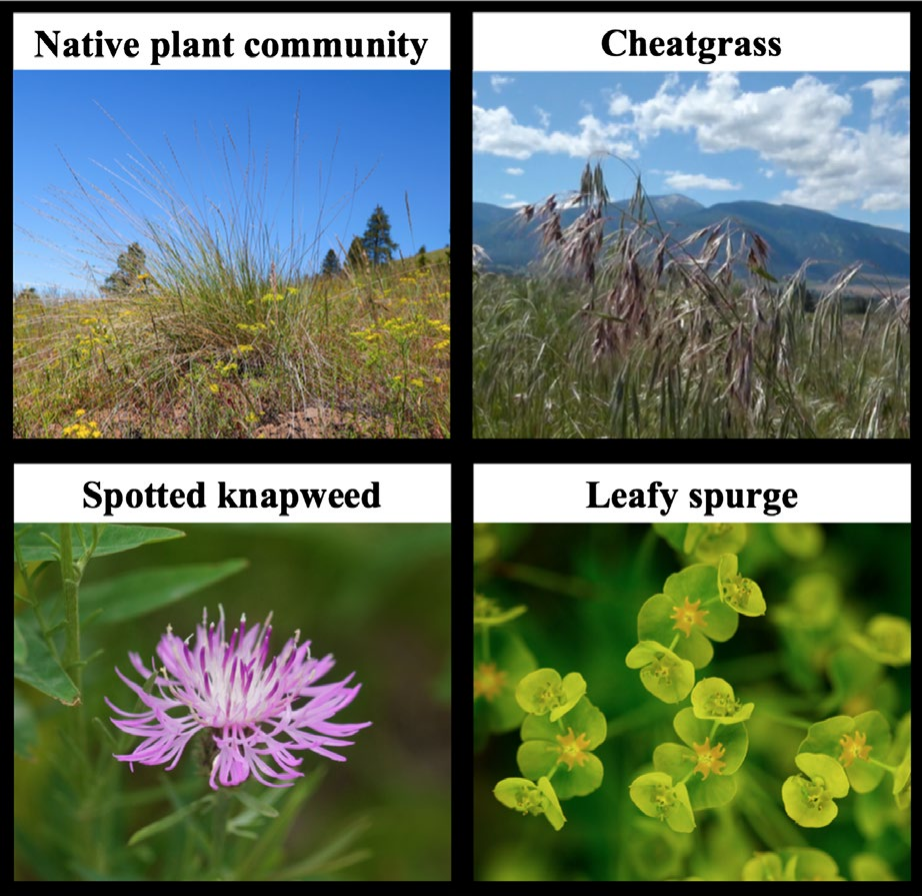
Photographs of a native plant community and three invasive species common to grasslands in the Intermountain West, USA. Photos courtesy of A. Ramsey and C. Spencer‐Bower

The consequences of plant invasion on ecosystem processes are often generalized from meta‐analyses and review articles that combine all invaders into one homogenous group (Ehrenfeld, 2003; Liao et al., 2008). While informative, these approaches may be biased by findings from heavily studied ecosystems. Also, even though invaders can share many attributes (e.g. high NPP), they often differ substantially in life histories, and those species‐specific differences may not be captured in meta‐analyses. In the intermountain west, for example, cheatgrass (*Bromus tectorum*), spotted knapweed (*Centaurea stoebe*), and leafy spurge (*Euphorbia esula*) invade grasslands and create persistent invasions (Figure 1). Yet cheatgrass is an annual grass that senesces early in the growing season (Mack & Pyke, 1983), whereas spotted knapweed and leafy spurge are perennial forbs that are active throughout the growing season (Messersmith, Lym, & Galitz, 1985; Sheley, Jacobs, & Carpinelli, 1998). Leafy spurge differs from spotted knapweed in that it has deeper roots, spreads through rhizomes, and exudes latex to defend against herbivores (Lym & Kirby, 1987; McLeod et al., 2016). The three species are highly invasive, produce more biomass, and are associated with higher soil nitrogen availability than native plants (McLeod et al., 2016). One possible reason for this is a faster turnover of litter (Fierer, Craine, McLauchlan, & Schimel, 2005; Wardle 2006), but to what extent litter decomposition rates and decomposer communities differ among these dissimilar invaders is unknown.

Many plants change the belowground microbial community in a way that increases decomposition rates, termed home‐field advantage (HFA) (Austin, Vivanco, González‐Arzac, & Pérez, 2014; Ayres et al., 2009; Elgersma, Yu, Vor, & Ehrenfeld, 2012). Home‐field advantages occur worldwide (Austin et al., 2014), but their role in plant invasion is not well known. It might be expected that if invasive plants, as a group, produce higher quality litter than native plants (Liao et al., 2008), decomposer communities may shift from oligotroph‐dominated to copiotroph‐dominated, which are organisms that thrive in low‐ and high‐nutrient environments, respectively (Fierer, Bradford, & Jackson, 2007). Indeed, one study found that litter from an invasive plant fostered a microbial community capable of faster decomposition of litter from both the invasive host and other plants (Elgersma et al., 2012). This could increase nutrient availability for the invader and generate a positive feedback, although more rigorous investigations of HFA in the field for multiple invaders are required to assess general patterns.

We compared decomposition rates and microbial communities associated with root and shoot litter of cheatgrass, spotted knapweed, and leafy spurge as well as bluebunch wheatgrass (*Pseudoroegneria spicata*), which is a native grass common to grasslands in the northern Rocky Mountains. We placed shoot litter on the surface and buried root litter from each plant species into replicated plant community types in a factorial design. Three research questions were addressed as follows: (1) Do decomposition rates differ among plant species? (2) Do microbial communities associated with shoot and root litter differ among plant species? (3) Is decomposition faster when litter is placed in a “home” community, that is, are invaders generating a HFA, which may further increase their capacity to invade?

## 2 MATERIALS AND METHODS

### 2.1 Site location and characterization

Our study site was located on MPG Ranch in Montana's Bitterroot Valley (46°40′48″N, 114°1′40″W, 1,024 m; mpgranch.com). The site was sprayed with broadleaf herbicides multiple times and continuously grazed from 1972 to 2007. Cattle were excluded 3 years prior to this study. We identified three locations that were a minimum of 1.5 km apart, each having four distinct plant communities that were dominated by cheatgrass, spotted knapweed, leafy spurge, and remnant native plants. The invasive communities were identified based on more than 50% coverage of target plants that were dead but standing from the previous season, whereas native communities were dominated by bunch grasses, and the cover of invasive species was less than 5%. Each invasion had been in place for more than 10 years, based on aerial photography and oral history records. Plant communities within each location had similar slope, elevation, and aspect and were within 100 m of each other, which reduced spatial heterogeneity among communities. In March 2010, we established plots (7 × 7 m) within each plant community at each location, for a total of 12 plots. One temperature data logger (Thermochron iButton, Maxim Integrated, San Jose, CA, USA) per plot was inserted in the soil (5 cm depth) on 9 April for continuous measurements. We quantified plant cover within four random 1 m^2^ areas per plot on 29 July 2010 after all plants had reached maturity (Table S1). Because soil bacterial communities are affected by soil pH (e.g. Fierer & Jackson, 2006; Rousk et al., 2010), we collected and pooled three soil samples (0–10 cm) within each plant community in all sites on 16 April, 6 June, and 7 October for pH analysis (AgSource Harris, Lincoln NE, USA).

### 2.2 Litter decomposition

To determine whether exotic plants alter decomposition rates and whether decomposers preferentially decompose litter they are most likely to encounter, we assessed the mass loss of litter when placed in either a “home” environment (e.g. cheatgrass litter placed in a cheatgrass community) or “away” environments (e.g. cheatgrass litter placed in a spotted knapweed community) in a full factorial design. We collected shoot and root litter from all plants from the three areas on 30 March 2010 and pooled litter across areas. Shoot samples consisted of dead material grown the previous year that was standing (stem, leaves, and seed heads) and cut into 5‐cm pieces and placed in 12 × 12 cm nylon mesh litterbag (1.5 mm openings). Shoot mass was adjusted for each species to minimize differences in litter volume. Litter mass equaled 4.0 g for leafy spurge, 3.5 g for spotted knapweed, and 2.0 g for cheatgrass and native. Native litter consisted of bluebunch wheatgrass, the most abundant species within native communities. We collected roots from underneath target plants that most likely varied in viability, except for cheatgrass where roots were all dead as it is an annual plant. Only fine roots (<1.5 mm) were included, and we used 1.75 ± 0.01 g of roots of each species and placed them in 8 × 8 cm mesh litterbags. We pinned shoot litterbags to the surface with lawn staples and buried root litterbags (5 cm depth) in all plant communities on 6 April. To control for losses due to handling, two replicate litterbags of each tissue type and plant species were placed in the field for 2 hr, retrieved, and weighed. We used this modified initial weight as the starting weight of all samples. Three litterbags per litter type were placed in all plots; one was retrieved after 3 months (27 July) and another after 6 months (18 October) for mass loss measurements, and one was retrieved after 6 months for molecular analyses of fungal and bacterial communities. Litterbags were opened and handled with care to minimize losses, rinsed in distilled water, and collected on a 250‐μm sieve and blotted dry. To assess mass loss, litter was dried at 65°C to constant weight. The litter used for molecular analyses was freeze‐dried. Litter quality (carbon, nitrogen, phosphorus, and lignin) was analyzed (Analytical Laboratory, University of California Davis) at incubation and 6 months after incubation on samples that were dried (65°C) and ground. Because of the quantity required and high cost of each litter analysis, we had only one replicate per treatment, which allowed for qualitative comparisons but precluded statistical analyses.

### 2.3 Microbial communities on litter

Fungal and bacterial communities were characterized using PCR and T‐RFLP, which includes fragment analyses of digested PCR products of whole fungal and bacterial communities (e.g. Mummey, Rillig, & Holben, 2005). The T‐RFLP method has been shown to quantify and characterize microbial communities without apparent bias (Cotton et al., 2014). DNA was extracted from 10 mg of milled and freeze‐dried litter using the CTAB‐based protocol of Gardes and Bruns (1993). Genomic DNA from the whole fungal community was amplified using PCR and fluorescently labeled ITS1f‐FAM and unlabeled ITS4 primers (White, Bruns, Lee, & Taylor, 1990) on optimized template concentration (1:100 for most samples). We followed these thermocycling parameters: 2 min at 95°C, 32 cycles of 30 s at 95°C, 1 min at 56°C, and 2 min at 72°C with a final extension phase of 5 min at 72°C. We amplified genomic DNA of bacteria using the fluorescently labeled 27f‐FAM and unlabeled 907r primers on optimized template concentration (1:10 for most samples). The thermocycling program was: 2 min 95°C, 30 cycles of 30 s at 95°C, 1 min at 56°C, and 2 min at 72°C with a final extension phase of 5 min at 72°C. We quantified the product and verified length using agarose gel electrophoresis. DNA concentration was standardized among samples based on band intensity prior to digestion. Purified fungal PCR products (3 μl) were digested with Hinf1 (2.5 U), and bacterial PCR products (3 μl) were digested with Hha1 (4 U) in a buffer according to the manufacturer's instruction (New England Biolabs, Beverly, MA) for 5 hr at 37°C. One microlitre of *E. coli*, which was amplified with fluorescently labeled 27f‐HEX, was added to each sample prior to digestion to allow us to assess whether digestion was complete, because uncut product can complicate T‐RFLP analyses (Mummey et al., 2005). T‐RF sizes in each sample were analyzed at the Murdock Molecular Biology Facility at the University of Montana using an ABI 3100 automated capillary DNA sequencer with ROX‐1000 as a size standard. Total relative fluorescence of T‐RFLP profiles was standardized based on the number of relative fluorescence units (RFU) and peaks below 50 RFU were removed from further analyses.

### 2.4 Statistical analyses

All statistics were calculated in RStudio (version 0.99.484; R Core Team, 2013). To test differences in temperature and pH among plant communities, we ran a two‐way ANOVA with plant community and month as fixed factors and location of incubation (location) as a blocking factor (lme4 and lmerTest packages; Bates, Maechler, Bolker, & Walker, 2015; Kuznetsova, Brockhoff, & Christensen, 2015). Litter decomposition was based on the percent of mass lost at 3 and 6 months and was analyzed as a randomized split‐plot design with plant species of litter as a factor within the subplot of plant community and each location represented the whole plot. Given that individual litter bags were incubated for the two harvests, we treated each time interval and litter type (i.e. roots or shoots) as independent and ran four separate analyses (two harvests for shoots and roots, respectively). Mean comparisons were calculated with a Tukey post hoc test using the multcomp package (α = 0.05; Hothorn, Bretz, & Westfall, 2008). We tested for homogeneity with a Levene's test (car package; Fox & Weisberg, 2011) and plotted the residuals of the models to confirm normality. T‐RFLP data for fungal and bacterial communities were evaluated using permutational multivariate analysis of variance (perMANOVA; adonis; vegan package) with Bray–Curtis distances (Oksanen et al., 2007). The model included plant species (litter) and plant community as explanatory variables with location as the blocking factor. The root and shoot data were separated for the analysis because they were incubated in two different environments (i.e. belowground vs. aboveground), which would confound direct comparison. We also reran the analyses by replacing the plant species with its functional group (e.g. forb or grass). Nonmetric multidimensional scaling (NMDS) was used to visualize the data (metaMDS; vegan). Statistical code can be accessed in Supplemental Information 2.

## 3 RESULTS

Qualitative comparisons indicate that the invasive forbs tended to have higher quality litter than the grasses. Litter from leafy spurge contained more nitrogen than spotted knapweed, cheatgrass, and bluebunch wheatgrass litter (Table S2). Leafy spurge and spotted knapweed roots contained more phosphorous than cheatgrass and bluebunch wheatgrass litter, resulting in substantial differences in C/P ratios. Roots contained higher concentrations of both N and P relative to shoots and lignin increased relative to nitrogen over time.

Soil temperature differed among plant communities (*F* = 4.98; *p* = .004) and was lowest in leafy spurge communities (15.6 ± 1.0°C; seasonal mean ± *SE*), with increasing temperatures in spotted knapweed (16.7 ± 1.3°C), cheatgrass (17.6 ± 1.1°C), and native communities (17.6 ± 1.1°C). Not surprisingly, soil temperature also differed across the season (*F* = 107.09; *p* < .001; Fig. S1). Soil pH differed among plant communities (*F* = 4.10, *p* = .019) but not across the season, so we pooled pH values for each species. We observed the lowest pH in native communities (6.43 ± 0.07), with increasing pH values in cheatgrass (6.63 ± 0.10), leafy spurge (6.67 ± 0.05), and spotted knapweed communities (6.72 ± 0.08).

**Figure 2:**
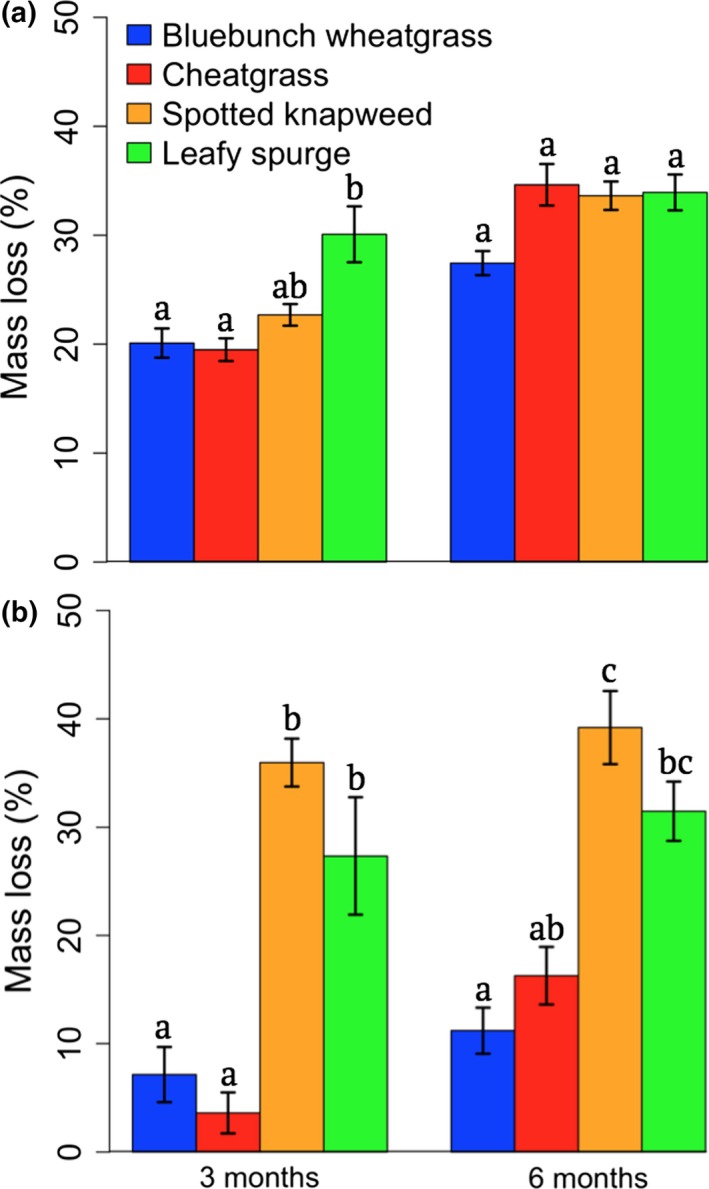
The mean proportional loss of litter (%) by plant species in which (a) shoots was pinned to the surface and (b) roots were buried (5 cm depth). Different letters above bars indicate statistical groupings (*p *<* *.05) based on a Tukey's post hoc test between plant species. The bluebunch wheatgrass community represented a diversity of native plants (Table S1). Error bars represent standard error

Leafy spurge shoots decomposed fastest and had lost 30% of their mass after 3 months, whereas spotted knapweed, cheatgrass, and bluebunch wheatgrass had lost about 20% (Table 1; Figure 2a). By 6 months, differences in decomposition had disappeared among shoots of all species (Table 1). There were large differences in root decomposition (Table 1; Figure 2b). Roots from spotted knapweed and leafy spurge had lost more than 25% of their mass after 3 months, whereas roots from cheatgrass and bluebunch wheatgrass had lost less than 10%. Those differences largely remained at 6 months, although mass loss of cheatgrass roots was no longer significantly different from leafy spurge (*p* = .076). The location of incubation did not influence the decomposition of either root or shoot litter, and there was no significant interaction between plant species and plant community, which would indicate a HFA.

**Table 1:** Results for the split‐plot design that tested differences in proportional loss of litter (%) for each plant species in each plant community of incubation at three and 6 months

	*df*	SS	*F*	*p*
Shoots				
3 months				
Plant species (S)	3	776.12	10.65	**<.001**
Plant community (C)	3	48.07	0.66	.584
S × C	9	278.86	1.28	.293
6 months				
Plant species (S)	3	401.21	8.36	**<.001**
Plant community (C)	3	8.32	0.17	.911
S × C	9	217.34	1.51	.201
Roots				
3 months				
Plant species (S)	3	8808.3	31.32	**<.001**
Plant community (C)	3	954.2	3.39	.074
S × C	9	890.9	1.06	.428
6 months				
Plant species (S)	3	6107.5	19.76	**<.001**
Plant community (C)	3	139.1	0.45	.719
S × C	9	362.5	0.39	.930

Bold indicates significant values (*P* ≤ 0.05).

**Figure 3:**
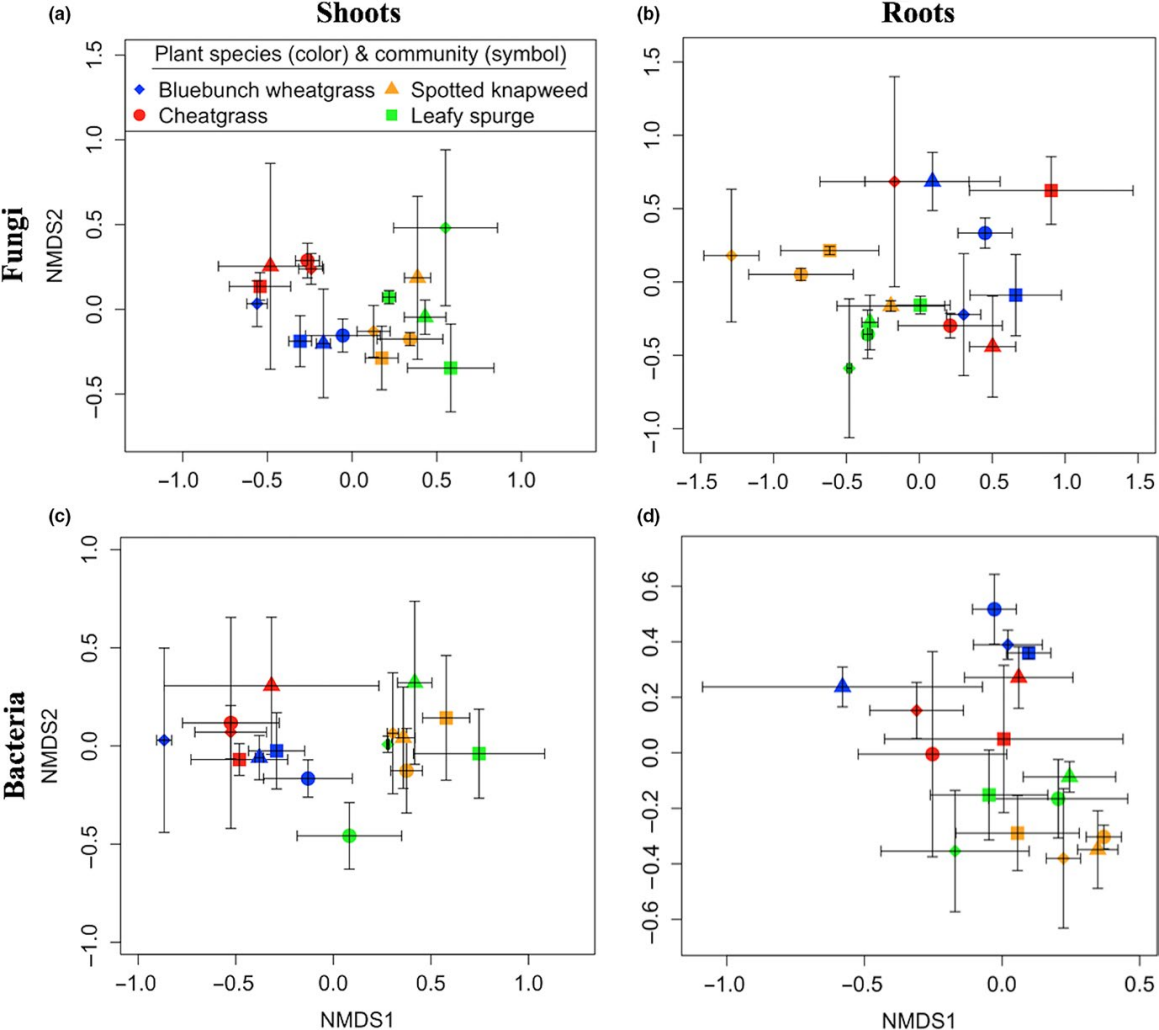
NMDS plots of fungi on (a) shoots and (b) roots and bacteria and on (c) shoots and (d) roots based on T‐RFLP profiles from litter of either native (bluebunch wheatgrass), cheatgrass, spotted knapweed, or leafy spurge. Colors represent the plant species of the litter, and the symbols represent the plant community in which the litter was placed. The bluebunch wheatgrass community represented a diversity of native plants (Table S1)

Fungal communities that colonized both shoot and root litter differed based on plant species and the plant community of incubation (Table 2; Figure 3a–b). When we re‐analyzed the data with respect to plant functional group, the fungal communities on shoot and root litter from forbs (spotted knapweed and leafy spurge) differed from those of grasses (bluebunch wheatgrass and cheatgrass) (Table S3), and in the case of shoots, differed based on the plant community of incubation. Bacterial communities that inhabited litter differed based on plant species (Table 2; Figure 3c–d) and whether the litter was from a forb or grass (Table S3) but did not differ among plant communities.

**Table 2:** Results from the perMANOVA analysis that used Bray–Curtis distances for both fungi and bacteria inhabiting shoots and roots of plant litter

	*df*	SS	*F*	*R* ^2^	*p*
Fungi					
Shoots					
Plant species (S)	3	2.02	4.70	0.24	**.001**
Plant community (C)	3	0.68	1.58	0.08	**.044**
S × C	9	1.39	1.08	0.16	.357
Residuals	31	4.44		0.52	
Total	46	8.52		1.00	
Roots					
Plant species (S)	3	2.72	3.70	0.21	**.001**
Plant community (C)	3	1.08	1.46	0.08	**.042**
S × C	9	2.48	1.12	0.19	.183
Residuals	27	6.62		0.51	
Total	42	12.90		1.00	
Bacteria					
Shoots					
Plant species (S)	3	1.97	5.09	0.29	**.001**
Plant community (C)	3	0.35	0.91	0.05	.549
S × C	9	1.02	0.88	0.15	.685
Residuals	27	3.48		0.51	
Total	42	6.82		1.00	
Roots					
Plant species (S)	3	1.73	4.67	0.28	**.001**
Plant community (C)	3	0.40	1.08	0.06	.369
S × C	9	0.90	0.81	0.14	.846
Residuals	26	3.20		0.51	
Total	41	6.24		1.00	

## 4 DISCUSSION

Previous work has shown that cheatgrass, spotted knapweed, and leafy spurge invasions associate with different bacterial and fungal communities (Gibbons et al., 2017; Lekberg et al., 2013). We show here that these species‐specific effects extend to litter, because microbial communities colonizing litter from these invaders differed significantly from each other and from a native bunchgrass. However, unlike earlier work showing that invasive plants often culture soil biota that promote their own growth (Callaway, Thelen, Rodriguez, & Holben, 2004; Klironomos, 2002), we found no evidence for a HFA when it came to decomposition rates; that is, litter did not decompose faster when placed in its home community. This may be because the effect of litter exceeded the effect of where the litter was placed, suggesting that bacterial and fungal decomposers can be widespread and that chemical and/or physical attributes of litter exert a strong habitat filter. Even so, some generalities based on plant functional group identity were apparent, because the two grasses harbored more similar microbial communities and decomposition rates than the forbs. This reiterates recent pleas to better incorporate a trait‐based approach in invasion biology (Bunn, Ramsey, & Lekberg, 2015; Meisner et al., 2014).

### 4.1 Decomposition rates differed among invasive plants

Litter decomposition is tightly driven by litter quality, which is often characterized by C:N ratios and lignin content (Silver & Miya, 2001; Zhang, Hui, Luo, & Zhou, 2008). A meta‐analysis of previously published data showed that exotic plants tend to decompose faster than native plants likely due to the high nutrient quality of the exotic plants (Liao et al., 2008). We found partial support for this because roots from spotted knapweed and leafy spurge decomposed faster than bluebunch wheatgrass roots (Table 1; Figure 2b). However, the slow decomposition rate of cheatgrass roots shows that generalizations about invasive plants do not always apply. Further, the differences in decomposition rates of roots depended not on whether they were native or exotic, but whether they were a forb or a grass. Roots from the two invasive forbs, spotted knapweed and leafy spurge, tended to have higher phosphorus content than roots from cheatgrass and bluebunch wheatgrass (Table S2), which may explain the disparity in decomposition rates because higher quality root litter tends to decompose faster (Silver & Miya, 2001; Zhang et al., 2008). The limited number of invaders included in the study clearly limits broad generalizations, but different decomposition rates have been shown depending on plant traits (Cornwell et al., 2008) and reinforce recent suggestions that plant life form should be included when analyzing the impacts of specific exotic plants (Meisner et al., 2014).

Shoots from leafy spurge decomposed faster than shoots from all other plants at 3 months, but at 6 months, there were no significant differences in decomposition (Table 1; Figure 2a). Like the decomposition of roots, the decomposition of shoots largely depends on the nitrogen and phosphorus content (Cornwell et al., 2008; Parton et al., 2007). Leafy spurge had more nitrogen content in its shoots than all other species, suggesting that at 3 months, the nutrient quality of litter may have influenced the different decomposition rates among species (Table S2).

Two important abiotic conditions that influence decomposition are soil moisture and temperature. Differences in these conditions were kept minimal between plant communities because we chose sites that were close in proximity to each other and shared similar aspect, slope, and elevation. However, plants can shade soils, which changes soil temperature and moisture, both of which influence decomposition rates (Köchy & Wilson, 1997). Cheatgrass communities had warmer (Fig. S1), and potentially drier soils, whereas leafy spurge communities had cool, and possibly wetter soils, yet these differences in abiotic conditions among plant communities did not lead to different decomposition rates. Although ultraviolet radiation contributes to the decomposition of shoot litter in grasslands as well (Austin & Vivanco, 2006; Parton et al., 2007), it likely did not drive differences observed here, because the plant community where litter was placed did not influence decomposition rates of shoots.

### 4.2 Bacterial and fungal communities differed among plant species

The nutritional makeup of litter can drive the structure of decomposer communities (Bray, Kitajima, & Mack, 2012; Cline & Zak, 2015; Purahong et al., 2016; Voříšková & Baldrian, 2013). We found that the forbs generally had greater nitrogen and phosphorus content than grasses (Table S2) while also harboring different decomposer communities (Table S3). As litter decomposes, r strategists (copiotrophs) use labile matter, and when only recalcitrant matter remains, k strategists (oligotrophs) become dominant (Dilly, Bloem, Vos, & Munch, 2004). Gibbons et al. (2017) found that spotted knapweed and leafy spurge shifted the bacterial communities in soils toward copiotrophs. This suggests that certain phyla of bacteria can become enriched in response to the litter used in this study.

One interesting finding was that decomposer communities depended more on the species of plant litter than the plant community of incubation (Table 2). Two different processes could explain this. First, all plant communities may harbor a diversity of fungal and bacterial species where a subset of the community colonizes litter depending on nutrient quality. This suggests that the characteristics of litter provide a strong habitat filter for these decomposers. An alternative explanation is that these bacteria and fungi occurred as endophytes that changed to saprophytic strategies upon senescence or harvest of plant tissues (Kembel & Mueller, 2014; Omacini, Chaneton, Ghersa, & Otero, 2004; Voříšková & Baldrian, 2013). While this could partly explain the strong effect of plant species, the dependence of fungal communities on the plant community of incubation (Table 2) suggests that the litter was at least partially colonized at the site of incubation. Bacterial endophytes also could have been present, however, the amount of bacterial DNA on litter at the onset of decomposition can be small (Dilly et al., 2004), suggesting that endophytic bacteria may not strongly influence initial decomposition.

Fungal communities did not restructure based on the nutritional quality of litter alone because they differed based on the plant community of incubation, showing that these persistent plant invasions created legacies of fungal communities that resisted change upon the introduction of new plant species. This supports previous work where microbial communities persisted even after the host litter had been removed and new litter was introduced (Elgersma, Ehrenfeld, Yu, & Vor, 2011; Elgersma et al., 2012). However, bacterial communities on litter did not depend on the plant community of incubation (Table 2), suggesting that they may rapidly restructure to the litter they encountered in an absence of a strong legacy effect. The relative importance of legacy effects and nutrient quality on decomposition rates and decomposers of invasive plants is unresolved but likely depends on plant species and duration of the invasion (Elgersma et al., 2011).

### 4.3 Home‐field advantage

The term HFA implies a positive effect where litter decomposes faster in its local environment, but effects are not always positive, suggesting HFA is context‐dependent (Freschet et al., 2012; Veen et al., 2015). Freschet et al. (2012) proposed that HFA is just a facet of a more comprehensive hypothesis called the substrate quality–matrix quality interaction (SMI) where HFA effects are greatest when litter quality is strongly dissimilar from the litter matrix associated with a site. For example, high‐quality litter placed in a matrix of high‐quality litter would decompose rapidly, but in contrast, it would decompose slower when placed in a matrix of lower quality litter (Freschet et al., 2012). The influence of HFA or the SMI in the decomposition of exotic plants is not well studied, although one study found that an invasive shrub changed the microbial communities in a way that increased decomposition rates (Elgersma et al., 2012). Here, we expected the strong dissimilarities in the nutrient quality of litter and long established plant communities (≥10 years) to change the decomposer community in a way that created HFAs based on the SMI hypothesis (Elgersma et al., 2012; Freschet et al., 2012; Strickland et al., 2009). For instance, decomposers within cheatgrass and native plant communities may specialize on recalcitrant matter, whereas decomposers in spotted knapweed and leafy spurge communities may specialize on labile matter. However, we did not observe a HFA even though each plant species reshaped the microbial communities and forbs tended to decompose faster than grasses.

The lack of an HFA effect may be explained by an insufficient disparity between the quality of litter (e.g. N:P) commonly found in each plant community where litter was incubated. In a meta‐analysis by Veen et al. (2015), they observed stronger HFA effects for forest–grassland transplants than for grassland–grassland transplants, which were likely driven by the SMI hypothesis. That finding, taken together with this study, further suggests that HFA effects in grasslands may be minimal. It is important to note that HFA effects can be subtle and often influence decomposition rates by <10% (Veen et al., 2015), so our small sample size may have precluded the detection of a minor change in decomposition rates.

## 5 CONCLUSIONS

By incubating native and invasive plant litter in the field, we determined that the high nutrient availability often observed in plant invasions may be driven in part by rapid decomposition of exotic plant litter. However, the substantial differences in decomposition of roots between cheatgrass and the two invasive forbs also indicate that generalizations do not apply to all invaders. Even though litter can culture a plant‐specific microbial community and fungi can persist when a novel litter is introduced, decomposition rates often did not differ based on whether the litter was placed in home or away soils. Overall, we show that exotic plants that are common to the same ecosystem may use different strategies toward creating successful invasions.

## CONFLICT OF INTEREST

The authors declare no conflict of interests.

## AUTHOR CONTRIBUTIONS

YL, DM, and PR designed the study. MM, YL, DM, and AR collected and analyzed the data. MM and YL led the writing of the manuscript, and all authors contributed critically to the drafts and gave final approval for publication.

## DATA ACCESSIBILITY

Data associated with this study will be deposited at datadryad.org.
