# Supplementary material for: Do invasive plants structure microbial communities to accelerate decomposition in intermountain grasslands?
Source: Ecol Evol. 2017 Nov 21;7(24):11227–35. doi: 10.1002/ece3.3608 (PMC5743483; doi:10.1002/ece3.3608)
Supplement: Supplementary file 1 [file ECE3-7-11227-s001.docx]

**Table S1**. GPS coordinates, weed abundance (based on percent cover in July, 2010) and plant species in each plot (dominant plant species in native plots are underlined).

| Area | Plant community | GPS coordinates | Weed abundance  Mean (%) ± s.e. | Plant species ID |
| --- | --- | --- | --- | --- |
| North | Bluebunch wheatgrass | 46°42’068’’ N, 114°01’339’’ W | 0 | *Hesperostipa comata, Pseudoroegneria spicata, Bouteloua gracilis, Gallardia aristata, Antennaria rosea, Poa compressa, Artemisia tridentate, Lupinus sp.* |
|  | Cheatgrass | 46°42’095’’ N, 114°01’340’’ W | 43 ± 7.5 | *Poa compressa, Verbascum blattaria, Sisymbrium altissimum* |
|  | Spotted knapweed | 46°42’112’’ N, 114°01’326’’ W | 39 ± 6.6 | *Poa bulbosa, Bromus tectorum (<5%)* |
|  | Leafy spurge | 46°42’081’’ N, 114°01’353’’ W | 91 ± 3.1 | *Lepidium latifolium, Sisymbrium altissimum Bromus tectorum (<5%)* |
| Orchard | Bluebunch wheatgrass | 46°41’342’’ N, 114°00’764’’ W | 5 ± 2.9 (cheatgrass) | *Pseudoroegneria spicata, Hesperostipa comata, Gallardia aristata, Lupinus sp, Balsamorhiza sagittata, Erigeron sp.* |
|  | Cheatgrass | 46°41’334’’ N, 114°00’720’’ W | 65 ± 12 | *Poa bulbosa*, *Balsamorhiza sagittata* |
|  | Spotted knapweed | 46°41’336’’ N, 114°00’782’’ W | 24 ± 9.4 | *Poa bulbosa, Gallardia aristata, Lupinus sp., Purshia tridentate, Tragopogon dubius, Bromus tectorum (28%)* |
|  | Leafy spurge | 46°41’352’’ N, 114°00’738’’ W | 60 ± 4.1 | *Poa bulbosa* |
| Woodchuck | Bluebunch wheatgrass | 46°40’768’’ N, 114°00’145’’ W | 0 | *Festuca ovina, Pseudoroegneria spicata, Aristida sp., Hesperostipa comata, Astragalus sp., Heterotheca villosa* |
|  | Cheatgrass | 46°40’663’’ N, 114°00’011’’ W | 38 ± 14 | *Festuca ovina, Poa compressa*, *Hesperostipa comata* |
|  | Spotted knapweed | 46°40’681’’ N, 114°00’006’’ W | 24 ± 7.5 | *Heterotheca villosa, Antennaria rosea, Poa compressa, Festuca ovina* |
|  | Leafy spurge | 46°40’773’’ N, 114°00’194’’ W | 24 ± 4.3 | *Hesperostipa comata* |

**Table S2.** Litter quality of plant species, roots vs. shoots, at zero and six months.

**Table S3.** Results from the perMANOVA analysis that used Bray-Curtis distances for both fungi and bacteria inhabiting plant litter. The plant species factor was replaced with functional group which represents either forb or grass.

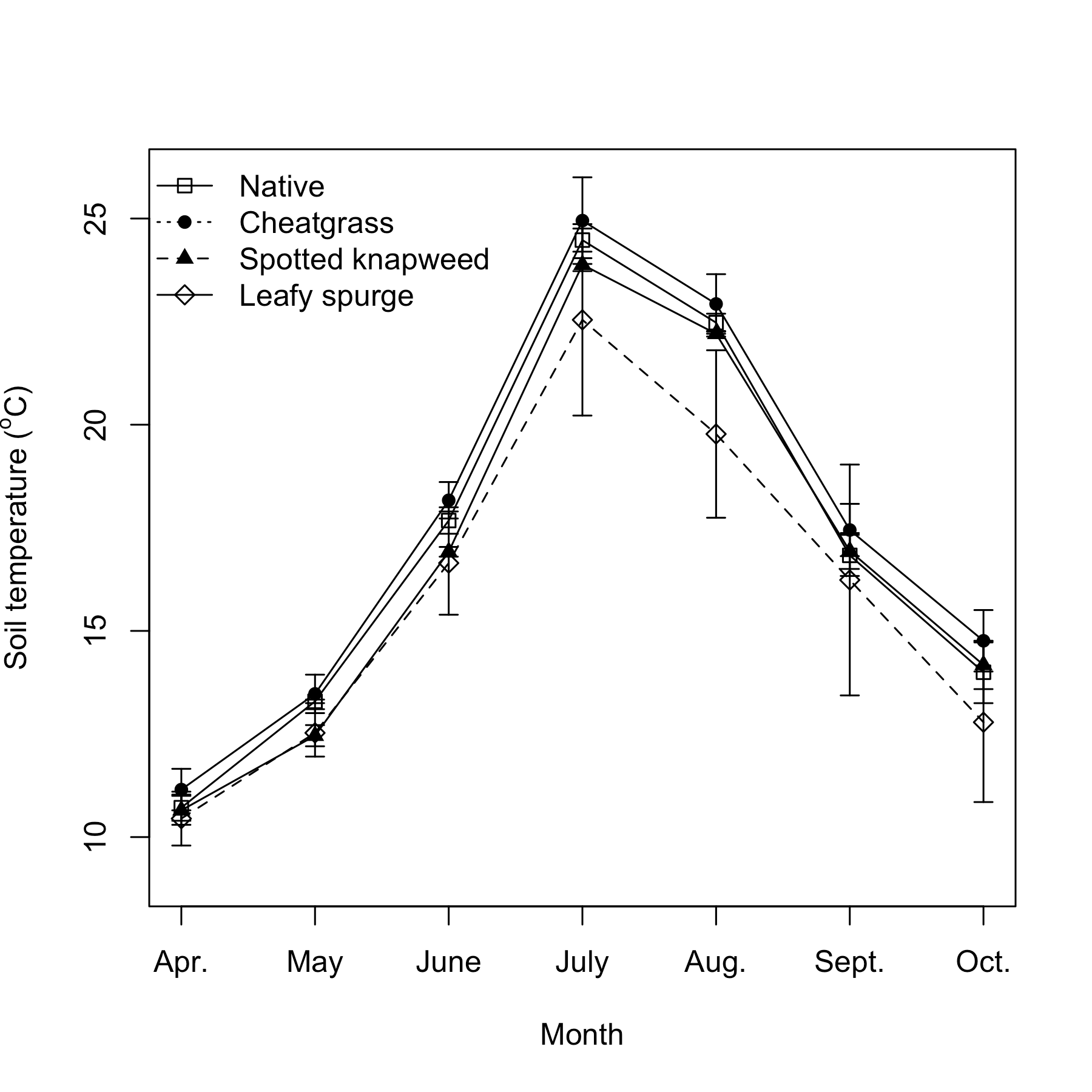


**Figure S1.** Monthly temperatures (°C) of soil in each plant community across the season (mean ± standard error).
